# Effects of Substituting Sedentary Behavior with Light-Intensity or Moderate-to-Vigorous Physical Activity on Obesity Indices in Adults: A Prospective Short-Term Follow-Up Study

**DOI:** 10.3390/ijerph182413335

**Published:** 2021-09-03

**Authors:** Bárbara de Barros Gonze, Thatiane Lopes Valentim Di Paschoale Ostolin, Evandro Fornias Sperandio, Rodolfo Leite Arantes, Marcello Romiti, Victor Zuniga Dourado

**Affiliations:** 1 Department of Human Movement Sciences, Federal University of São Paulo (UNIFESP), Santos, São Paulo 11015-020, Brazil; barbara.gonze@unifesp.br (B.d.B.G.); sperandio@unifesp.br (E.F.S.); victor.dourado@unifesp.br (V.Z.D.); 2 Angiocorpore Institute of Cardiovascular Medicine, Santos, São Paulo 11065-910, Brazil; arantes.r@uol.com.br (R.L.A.); marcello.romiti@angiocorpore.com.br (M.R.)

**Keywords:** obesity, body weight changes, sedentary lifestyle, accelerometry, isotemporal substitution

## Abstract

Background: Sedentary behavior (SB) is an independent risk factor for cardiovascular diseases. We hypothesized that there may be benefits of replacing SB with light-intensity (LIPA) and moderate-to-vigorous (MVPA) physical activity. Substituting SB with LIPA and MVPA might be associated with body composition changes. Methods: We assessed body weight, body mass index (BMI), fat body mass (FBM), and physical activity level, as well as one-year changes, in 780 adults (EPIMOV Study). Results: We analyzed into 10-min blocks SB, LIPA, MVPA, and total wear time. After 14 ± 3 months of follow-up, there were 242 completed procedures. We reallocated time spent in SB to LIPA or MVPA and assessed cross-sectional and prospective associations with the outcomes using isotemporal substitution models. In cross-sectional analysis, substituting 10-min blocks of SB with MVPA led to significant decreases of 1.23 kg in body weight, 0.30 kg/m² in BMI, and 0.38% in FBM. 10-min blocks substituting SB with LIPA produced significantly lower body weight (1 kg) and BMI (0.1 kg/m²) values. In longitudinal analysis, reallocating SB to MVPA was only associated with FBM decline (−0.31%). Conclusions: Substituting SB with MVPA is associated with significant improvement in obesity indices in both cross-sectional and follow-up. Replacing SB with LIPA produced a less consistent impact.

## 1. Introduction

Obesity is a growing public health problem worldwide and is commonly associated with several comorbidities [1]. One of the main triggers of obesity is the energy imbalance between consumption and caloric expenditure [2].

The increase in energy expenditure provided by physical activity (PA), as well as its health benefits and its inverse relationship with obesity and mortality, are already well described in the literature [3]. However, the research on sedentary behavior (SB), which is defined as “any waking behavior characterized by an energy expenditure ≤1.5 metabolic equivalent of task (METs) while in a sitting, reclining or lying posture” [4], has increased in the past few years, but still presents several gaps, especially when it is considered that even terminology consensus is recent [5,6,7]. Previous studies, including a systematic review and an overview, suggested that SB is related to increased cardiovascular risk, cancer, type 2 diabetes, metabolic syndrome, and all-cause mortality in adults [8,9,10]. Although some studies suggested a positive association between SB and obesity, it is controversial whether these deleterious effects are merely a consequence of PA time that has been substituted by time spent in SB [11,12,13,14].

Isotemporal substitution modeling takes into account that the total time in a day is finite, and therefore the total time spent in each PA spectrum, as well as the total accelerometer wear time, are interdependent [15]. Thus, this statistical approach allows the evaluation of the effect of substituting a block of time spent in one type of activity with another. Unfortunately, most of the previous studies using isotemporal substitution modeling were cross-sectional, including children and adolescents in high-income countries [9,10,11,12,13,14,15,16]. Few studies have explored this topic in adults, particularly in a prospective follow-up study. Obesity is usually classified by body mass index (BMI) [17]. The use of fat body mass as a percentage (%FBM), which is a more accurate marker of obesity [18], is scarce in the literature. It is worth mentioning that both SB and PA levels can be measured by questionnaires and wearable devices. In comparison to questionnaires [19], the advantage of accelerometers lies in the objectivity of the measurement [20].

Therefore, we conducted a prospective short-term follow-up study to test the hypothesis that replacing SB with light-intensity (LIPA) and moderate-to-vigorous physical activity (MVPA) would be associated with improvements in body weight, BMI, and %FBM in asymptomatic adults. We also assessed the effects of substituting SB for LIPA and MVPA in a cross-sectional design.

## 2. Materials and Methods

### 2.1. Selection of Subjects and Study Design

This study is part of the Epidemiology and Human Movement Study (the EPIMOV study), a cohort study that started in late 2013. The monitoring of participants in the EPIMOV study focused on cardiorespiratory diseases and locomotor disturbances. We have included only adults above 18 years old and free from cardiopulmonary diseases and locomotor disturbances at baseline, or other conditions diagnosed with an electrocardiogram or screening indicating that the participant would probably not be able to perform physical exercises safely. Participants were recruited through social networks and ads placed in local universities, magazines and newspapers.

Study assessments were carried out over two days, a week apart. Participants also contributed a blood sample in between the two visits at their earliest convenience. In the first visit, participants underwent general health screening, anthropometrics, spirometry, and cardiopulmonary exercise testing. Those participants with spirometric abnormalities suggesting obstructive lung disease were excluded from the present study. A forced vital capacity maneuver was performed using a suitably calibrated spirometer (Quark PFT, COSMED, Pavona di Albano, Italy) according to the criteria established by the American Thoracic Society [21], before and after inhalation of 400 micrograms of salbutamol [22,23]. At the end of the first assessment, participants were informed about the use of the triaxial accelerometer for the subsequent seven days.

In the second visit, they returned the accelerometer, answered the international physical activity questionnaire (IPAQ), and underwent assessments of body composition (bioelectrical impedance). After 14 ± 3 months had passed since the first evaluation, all participants were invited by telephone to repeat the entire research protocol.

Among the participants in the EPIMOV study, we included in the present study those who made correct and adequate use of the accelerometer (4 to 7 days, for at least 10 h/day) and properly followed the evaluation protocol required in the bioelectrical impedance analysis (i.e., adherence to test guidelines and protocols). The results of 780 participants at baseline, and 242 with a second assessment after follow-up, were analyzed.

We obtained written informed consent from all participants and the Ethics Committee at the local university approved the EPIMOV study (#186.796/2013).

### 2.2. Clinical Evaluation

Initial clinical evaluation included the history of health disorders and prior use of medications. We also investigated the presence of self-reported previous medical diagnosis of the main risk factors for cardiovascular disease, including older age (≥45 years for males and ≥55 for females), hypertension, diabetes/hyperglycemia, and dyslipidemia/hypercholesterolemia. Family history of premature coronary heart disease was defined as myocardial infarction or sudden death before 55 years of age in father or another male first-degree relative, or before 65 years of age in mother or other female first-degree relative. We also asked participants about current smoking.

### 2.3. Anthropometrics and Body Composition

We measured body mass and height on a scale equipped with a stadiometer (TOLEDO, São Paulo, Brazil), and then calculated the BMI [24].

We evaluated body composition and obtained %FBM using four-pole bioelectrical impedance (310E, BIODYNAMICS, Detroit, MI, USA). We instructed all participants to urinate at least 30 min before the test, remove all metallic objects from the body, not to use diuretics, keep fasting for at least four hours, not to drink alcohol for at least 48 h and remain for 10 min in the supine position at rest before the examination. The entire protocol followed the manufacturer’s instruction manual. We classified obesity by using %FBM values as being >30% for females and >20% for males [13] and from the BMI as ≥30 kg/m² [25]. 

### 2.4. Measures of Physical Activity and Sedentary Behavior

We performed this evaluation with a previously validated triaxial accelerometer (ActiGraph GT3X+, MTI, Pensacola, FL, USA) [26,27,28]. Accelerometers were given personally to the volunteers with instruction for proper wear. The participants wore the accelerometer on the dominant hip and were instructed to complete seven consecutive days of the assessment during usual waking hours. We instructed the participants to use the accelerometer until bedtime, except in the shower and in water-related activities. To be considered valid, days of data collection required at least 10 h of continuous monitoring, starting upon waking up. A minimum of 3 weekdays and 1 weekend day was necessary for inclusion in the final analysis. We considered non-wearing time as an interval of zero counts for 60 or more minutes. Both non-wearing time and the thresholds for the intensity of the physical activity were evaluated as previously described [29]. 

The total amount of sedentary behavior was considered based on minutes with counts less than 100 counts per minute (cpm), which represents <1.5 METs of energy expenditure, including active and non-active sitting, lying, and standing. The thresholds for the intensity of the physical activity were as follows [22]: very light (100–759 cpm); light (760–1951 cpm); moderate-to-vigorous (>1951 cpm). The activities in these thresholds included household activities (e.g., laundry, dishwashing, moving a small load and vacuuming) and locomotor activities (e.g., slow, normal or brisk walking, walking while carrying a bag, jogging or running, among others). We considered physically inactive participants as those with less than 150 min of MVPA per week [30,31]. We evaluated SB, LIPA, and MVPA at baseline and second visit.

The measurements were calculated as minutes/day considering the total wear time and wear time per calendar day as well as percentage of total time. 

### 2.5. Statistical Analysis

We performed statistical analyses using the SPSS package, version 23 (SPSS IBM Corp., Armonk, NY, USA). We used paired t-test for assessing changes in obesity and PA variables over time.

We investigated the effects of replacing SB by LIPA and MVPA by using isotemporal substitution modeling. Total accelerometer wear time, as well as the times spent in SB, LIPA, and MVPA, were converted into 10-min blocks. We chose 10-min blocks because this represents the minimum amount of time in which activities should be accumulated to meet the World Health Organization PA recommendations [31]. Thus, we assessed the association between time units of 10 min of SB, LIPA and MVPA with body mass, BMI, and %FBM using multiple linear regression.

Subsequently, we performed three sets of multiple linear regression analysis, so-called single models. We included all cardiovascular risk factors (age, sex, arterial hypertension, dyslipidemia, diabetes, and smoking) as covariates in all three statistical analyses. In the first model, we investigated the isolated impact of each PA intensity (SB, LIPA, and MVPA) on obesity indices (body mass, BMI, and % FBM). In these models, we used each obesity index as outcomes, one of three activity intensities separately (SB, LIPA, and MVPA) and all covariables. However, we did not adjust for the other activity intensities and for the total accelerometer wear time.

(1) Obesity index = b0+ b1 * (SB or LIPA or MVPA)+(b2 to b7) * (respective covariate)

In the second model, namely partition, we used obesity indices as outcomes and included all three PA categories (SB, LIPA, and MVPA) and the covariables simultaneously. We kept total wear time out of these models. With no adjustment for the total wear time, we assumed that each of the PA intensities was added to the model, rather than replaced. Accordingly, the model estimates each time component while keeping the others constant.

(2) Obesity index=b0+b1 * (SB)+ b2* (LIPA)+ b3* (MVPA)+(b4 to b9)* (respective covariate)

Finally, we performed the isotemporal substitution model to investigate the impact of replacing SB with LIPA and MVPA. We adjusted the model for all three PA categories as well as the total wear time and covariates. We assumed that any time spent in one type of PA causes a consequent isotemporal substitution of another kind of activity, since the total time is kept constant.

For example, to evaluate the impact of substituting 10 min of SB with 10 min of MVPA or LIPA, we removed SB from the model, maintaining LIPA, MVPA, total wear time and covariates. Thus, the coefficients of MVPA and LIPA represent the impact of reallocating SB to MVPA and LIPA in obesity indices as follows:

(3) Obesity index = b0+b2 * (LIPA)+ b3 * (MVPA)+(b4 to b9) * (respective covariate)+ b10 * (total wear time)

All statistical methods mentioned above were also applied using changes in body mass, BMI, and %FBM over time. In this case, we also adjusted all follow-up models for the baseline values of obesity indices. We verified the linearity of the relationships, as previously recommended, for isotemporal substitution modelling [15,32].

The sample size was calculated by the number of independent variables of interest for inclusion in multiple regression models, e.g., SB, LIPA, MVPA, age, sex, arterial hypertension, dyslipidemia, diabetes, smoking, and total accelerometer wear time. In the follow-up analyses, multiple regression models were also adjusted for body mass, BMI or %FBM obtained in the first evaluation. Considering 15 observations for each of the predictors, we found at least 150 subjects for inclusion in the study and 165 individuals for follow-up analyses.

## 3. Results

Of the 780 study participants, 242 (62 males) completed the first reassessment after the follow-up. At baseline, participants wore the accelerometer for 884 ± 76 min/day. The percentages of the total time in PA categories were: 73.2%, 21.6%, and 5.0% in SB, LIPA, and MVPA, respectively. The proportion of physical inactivity was 30%.

The initial evaluation of the total sample consisted mainly of middle-aged and overweight females (Table 1). The prevalence percentages of arterial hypertension, dyslipidemia, diabetes, and smoking at baseline were similar to those obtained after one-year follow-up, as well as the levels of PA (Table 1 and Table 2).

After the follow-up, we found significant absolute changes in MVPA and %FBM. We did not find significant absolute changes in either SB and LIPA or body mass and BMI (Table 2).

After cross-sectional single models, SB was poorly associated with BMI, body mass and %FBM. On the other hand, we found significant negative correlations between MVPA and body mass, BMI and %FBM. After cross-sectional partition models, only MVPA remained a significant determinant of obesity indices. After the follow-up, we found significant correlations between SB and MVPA with %FBM after single models. Regarding the longitudinal partition models, only SB was selected as a significant predictor of the absolute change in %FBM (Table 3).

The cross-sectional isotemporal substitution analysis showed that substituting 10-min blocks of SB with MVPA was significantly related to decreases in body weight, BMI and FBM. Substituting 10-min blocks of SB with LIPA produced significantly lower values of body weight and BMI. We found no effects of substituting SB with LIPA on FBM. As for the longitudinal analysis, we observed that replacing SB with MVPA was only associated with a significant decline in FBM (Table 4).

## 4. Discussion

In the present study, both cross-sectional and longitudinal designs were used to assess the effects of replacing SB with LIPA and MVPA on body mass, BMI, and %FBM. To our knowledge, few studies have addressed isotemporal substitution modeling to investigate prospectively the effects of substituting different PA categories on obesity indices [33,34,35], particularly in asymptomatic adults, by using triaxial accelerometers and adjusted by cardiovascular risk. We found that replacing 10-min blocks of SB with the same amount of time of LIPA or MVPA was prospectively associated with improvement in obesity indices. Despite the short follow-up period, we observed a decrease in body composition, which is also an important finding, especially regarding public health and the development of new strategies to promote PA, reduce SB, and control body weight.

Previous studies [36] showed that a higher SB is associated with an increased risk of cardiovascular disease and all-cause mortality. In addition, the SB has emerged as an independent risk factor for incident heart failure, regardless of MVPA [37]. Although the literature fully addresses the association between SB and PA with obesity, knowledge is scarce as to whether SB is an independent risk factor for obesity or rather just a consequence of the substitution of MVPA [38,39,40]. 

In our study, SB presented non-significant correlations with obesity indices after single and partition models. MVPA showed more consistent results [41]. Thus, our results reinforce current literature for both SB and MVPA findings. A recent meta-analysis showed no significant associations between SB and BMI, body weight, and waist circumference, which agrees with our main findings [13]. Although unexpectedly, it is reasonable that SB did not present significant associations with obesity indices, since being physically active can alter the negative effects of SB in cardiometabolic health, including BMI and waist circumference [12]. Moreover, despite the non-significant associations, Campbell et al. [13] showed that, with higher SB, the higher the odds of becoming overweight or obese which may be explained by the large heterogeneity of the studies. Lastly, this also can be attributed to behavior patterns and possibly mediation effects played by PA, as already explored in a previous study [42]. A large study [38] showed that decreases of as little as 6 min/day in MVPA were associated with an increased risk of obesity, which was not observed for SB. Previous studies [38,40,43] have indicated that objectively measured SB has little or no association with BMI. Conversely, MVPA presented a consistent correlation with BMI and lean mass [38,39,40,44,45]. These findings may be attributed to the higher energy expenditure generated by MVPA, resulting in decreased body mass [2]. In fact, guidelines for PA advocate MVPA to achieve health benefits [32]. However, a recent study observed that even leisure time PA has been shown to have positive effects on cardiometabolic health [46]. Unfortunately, there is no consensus regarding the amount of SB that should be avoided to reduce the risk of obesity and other major health problems.

We found significant benefits of replacing a small amount of SB with LIPA and MVPA on obesity indices. Our findings are consistent with previous studies, which observed that substituting SB with MVPA leads to a beneficial impact on body composition [16,33,34]. Despite methodological differences (e.g., using a wrist-worn accelerometer to measure SB and PA), we corroborate the results of Galmes-Panades et al. [33] which observed that replacing 30 min of SB with LIPA or MVPA decreases obesity indices (e.g., BMI, total fat, waist circumference, and visceral adiposity) in middle-aged subjects. Previously, Sardinha et al. [16] found that reallocating 15 or 30 min/day of SB to MVPA was positively associated with decreased obesity in children. Additionally, the isotemporal substitution of 30 min/day of academic activities or time spent using electronic devices for MVPA in children was associated with decreased BMI [47], while replacing one hour of SB with MVPA was positively associated with a lower %FBM [48]. Thus, SB did not prove to be an independent risk factor for obesity in the present study in agreement with those previously described, but rather its deleterious effects on health may have occurred due to the amount of MVPA time replaced with SB [49]. 

According to Grgic et al. [35] in a scoping review, studies that investigated reallocating movement-related behaviors were mostly cross-sectional with a wide variation in the amount of time replaced (e.g., from one minute to 120 min/day). Despite Danquah et al. [34] using similar procedures to our assessments, their study analyzed the substitution of 60 min spent sitting by standing at the workplace. The authors observed a decrease in body composition, but their findings were more expressive in cross-sectional than longitudinal analyses. In the present study, we can state that even a small amount of SB (e.g., 10 min/day) substituted for MVPA was sufficient to improve body composition in asymptomatic adults. However, our results agree with Danquah et al. [34], since the cross-sectional models lead to a decrease in all obesity indices (−1.23 kg in body weight, −0.30 kg/m^2^ in BMI, and −0.38% in FBM), while the longitudinal models reduce only FBM (−0.31%). Similar results were also observed by Hamer et al. [14], who found positive effects of replacing only 10 min/day of SB with MVPA on BMI (−0.39; from −0.54 to −0.24) and other metabolic risk factors in adults. 

Although our statistical approach is a theoretical intervention, the present study has some practical implications. We may affirm that even the substitution of small blocks of SB for the same amount of time of LIPA and MVPA were related to improved obesity indices and would be a more desirable strategy for decreasing obesity compared to strategies with a single focus on reducing SB. Thus, our results may be useful for designing more effective strategies to control body weight and prevent adult obesity pandemics, e.g., encouraging groups in the workplace to replace even a few minutes of SB with PA or stimulating the substitution of SB by PA in leisure-time. Accordingly, García-Hermoso et al. [41] observed that greater improvement in body composition was related to the replacement of 60 min of SB with MVPA in young subjects. Although interventions in the workplace were associated with decreased obesity [39,40], knowledge of the damaging effects of physical inactivity might not be sufficient to decrease this behavior in certain workers’ groups [50]. However, interventions in the workplace were associated with decreased obesity [51,52]. The social environment during physical exercises, as well as good nutritional behaviors, were also associated with increased consumption of healthy foods and PA levels. It is worth mentioning that setting small goals can be more easily attained than meeting PA recommendations, especially for subjects with chronic conditions or increased cardiovascular risk who largely benefit from lifestyle behavior change. However, we reinforce the need for setting goals for both SB and MVPA. Similar to our results, Ryan et al. [53] found that engaging in bouts equal to or higher than 10 min helps to control total cholesterol concentration and decrease triglyceride concentration in older adults. This agrees with our results, which becomes of special interest when we consider our findings of positive results in the decrease of obesity indices, even with small SB substitutions.

Some limitations of the present study should be considered. We did not evaluate other determinants of obesity, such as dietary and calorie intake. Additionally, we had a high loss to follow-up rate. Our short follow-up period is also a limitation. However, even after a short follow-up period, the benefits mentioned above may occur, which is an important point of our study. Using the triaxial accelerometer may lead to imprecise measurements of PA patterns in subjects that perform aquatic activities, wrestling, and cycling. However, our sample was exposed to a supportive environment for being active, since the region favors active transportation and has fewer weather condition impacts on PA level in comparison to regions with extreme weather conditions [54,55]. Additionally, the prevalence of physical inactivity and risk factors for cardiovascular disease are compatible with those found in the Brazilian population. Thus, some strengths of our research are worth noting. Among them, we used a classification of obesity based on %FBM and evaluated the levels of SB and PA using triaxial accelerometers. We were also unable to find previous prospective follow-up studies using isotemporal substitution modeling to assess the effects of reallocating SB to PA on changes in %FBM in asymptomatic adults.

## 5. Conclusions

We may conclude that SB has an inconsistent influence on the obesity indices in asymptomatic adults and its replacement with MVPA is associated with a significant improvement in body composition, even during a short follow-up. Studies with longer follow-up may confirm our results and clarify whether physical activity levels mediate the deleterious effects of SB.

## Figures and Tables

**Table 1:** General characteristics of the participants analyzed at baseline (*n* = 780).

Variables	n (%) or M ± SD
Age (years)	44.0 ± 14.5
Sex	
Males	287
Females	493
Body mass (kg)	76.8 ± 17.8
Body mass index (kg/m^2^)	28.6 ± 6.1
Fat body mass (kg)	24.6 ± 10.4
Fat body mass (%)	31.0 ± 8.5
Obesity	278 (35.6)
Arterial hypertension	139 (17.8)
Diabetes	83 (10.6)
Dyslipidemia	212 (27.2)
Smoking	90 (11.5)
Sedentary behavior (min/d)	650.9 ± 74.1
Light-intensity physical activity (min/d)	196.8 ± 49.3
Moderate physical activity (min/d)	40.5 ± 21.6
Vigorous physical activity (min/d)	1.8 ± 4.0

Data are presented as a mean ± standard deviation for continuous variables and as absolute count (%) for categorical variables.

**Table 2:** Absolute changes in obesity indices and physical activity intensities after the follow-up (*n* = 242).

Variables	Baseline	Follow-Up (14 ± 3 Months)	Absolute Change	*p*
Obesity indices				
Body mass (kg)	76.1 ± 17.1	76.3 ± 17.5	0.13 ± 5.52	0.702
Body mass index (kg/m^2^)	28.4 ± 6.0	28.5 ± 6.1	0.11 ± 1.84	0.348
Fat body mass (kg)	24.7 ± 11.0	26.3 ± 11.1	1.56 ± 6.40	0.000 *
Fat body mass (%)	31.0 ± 9.2	33.4 ± 9.2	2.40 ± 6.21	0.000 *
Physical activity intensity (min/d)				
Sedentary	653.4 ± 83.4	657.6 ± 86.8	4.17 ± 86.32	0.544
Light	199.3 ± 51.1	194.1 ± 57.4	−5.23 ± 42.88	0.127
Moderate	44.1 ± 21.1	38.9 ± 18.2	−5.23 ± 18.39	0.000 *
Vigorous	3.86 ± 8.00	3.61 ± 8.65	−0.25 ± 8.11	0.692
Moderate−to−vigorous	48.4 ± 24.4	42.8 ± 22.2	−5.65 ± 21.13	0.001 *

* *p* ≤ 0.05. Data are presented as mean ± standard deviation.

**Table 3:** Single and partition models assessing the correlations of physical activity intensities and obesity indices.

Variables	Body Mass (kg)	Body Mass Index (%)	Fat Body Mass (%)
Cross−sectional single models, B (SE)			
SB (10-min block/d)	−0.069 (0.127)	−0.044 (0.026)	−0.005 (0.029)
LIPA (10-min block/d)	−0.136 (0.221)	0.040 (0.041)	−0.044 (0.045)
MVPA (10-min block/d)	−1.264 (0.435) *	−0.284 (0.088) *	−0.357 (0.096) *
Cross−sectional partition models, B (SE)			
SB (10-min block/d)	−0.213 (0.139)	−0.066 (0.030)	−0.049 (0.033)
LIPA (10-min block/d)	−0.125 (0.238)	0.037 (0.047)	−0.033 (0.051)
MVPA (10-min block/d)	−1.418 (0.457) *	−0.369 (0.092) *	−0.388 (0.102) *
Longitudinal single models, B (SE)			
SB (10-min block/d)	−0.051 (0.044)	−0.007 (0.016)	0.116 (0.068)
LIPA (10-min block/d)	−0.014 (0.076)	−0.036 (0.032)	0.087 (0.119)
MVPA (10-min block/d)	0.000 (0.153)	−0.058 (0.063)	−0.269 (0.144) *
Longitudinal partition models, B (SE)			
SB (10-min block/d)	−0.067 (0.049)	−0.003 (0.020)	0.155 (0.076) *
LIPA (10-min block/d)	−0.055 (0.084)	−0.032 (0.035)	0.202 (0.130)
MVPA (10-min block/d)	−0.042 (0.161)	−0.047 (0.067)	−0.087 (0.250)

Cross-sectional analysis (*n* = 780); Longitudinal analysis (*n* = 242). * *p* ≤ 0.05. B (SE) represents coefficients (standard error). Single models: adjusted for physical activity intensities, i.e., sedentary behavior (SB), light-intensity physical activity (LIPA) or moderate-to-vigorous physical activity (MVPA), and covariates (age, sex, arterial hypertension, dyslipidemia, diabetes, and smoking). Partition model: adjusted for SB, LIPA, MVPA, and covariates mentioned above. Longitudinal analyses were also adjusted for values of obesity indices at baseline. SB, LIPA, and MVPA were calculated in 10-min blocks as well as total wear time.

**Table 4:** Effects of replacing sedentary behavior with light and moderate-to-vigorous physical activity on obesity indices.

10−Min Sedentary Behavior Substitution	Body Mass (kg)	Body Mass Index (kg/m^2^)	Fat Body Mass (%)
Cross−sectional isotemporal substitution models, B (SE)			
LIPA (10-min block/d)	−0.099 (0.230) *	−0.104 (0.044) *	0.018 (0.048)
MVPA (10-min block/d)	−1.237 (0.448) *	−0.303 (0.091) *	−0.383 (0.100) *
Longitudinal isotemporal substitution models, B (SE)			
LIPA (10-min block/d)	0.011 (0.083)	−0.004 (0.030)	0.037 (0.084)
MVPA (10-min block/d)	0.026 (0.161)	−0.039 (0.057)	−0.315 (0.149) *

* *p* ≤ 0.05. Models adjusted for 10-min blocks of LIPA, MVPA and total accelerometer wear time as well as for covariates (age, sex, arterial hypertension, dyslipidemia, diabetes, and smoking). The longitudinal analysis was also adjusted for baseline values of obesity indices at baseline.

## Data Availability

The datasets used and/or analyzed during the current study are available from the corresponding author on reasonable request.

## References

[1] Abelson P., Kennedy D. (2004). The obesity epidemic. Science.

[2] WHO (2015). Obesity and Overweight Factsheet from the WHO. http://www.who.int/mediacentre/factsheets/fs311/en/.

[3] Physical Activity Guidelines Advisory Committee (2008). Physical Activity Guidelines Advisory Committee Report: 2008.

[4] Sedentary Behaviour Research Network (2012). Letter to the editor: Standardized use of the terms “sedentary” and “sedentary behaviours”. Appl. Physiol. Nutr. Metab. Physiol. Appl. Nutr. Metab..

[5] Owen N., Healy G.N., Matthews C.E., Dunstan D.W. (2010). Too much sitting: The population health science of sedentary behavior. Exerc. Sport Sci. Rev..

[6] Tremblay M.S., Aubert S., Barnes J.D., Saunders T.J., Carson V., Latimer-Cheung A.E., Chastin S.F.M., Altenburg T.M., Chinapaw M.J.M., Sbrn Terminology Consensus Project Participants (2017). Sedentary Behavior Research Network (SBRN)—Terminology Consensus Project process and outcome. Int. J. Behav. Nutr. Phys. Act..

[7] Després J.P. (2016). Physical Activity, Sedentary Behaviours, and Cardiovascular Health: When Will Cardiorespiratory Fitness Become a Vital Sign?. Can. J. Cardiol..

[8] Young D.R., Hivert M.F., Alhassan S., Camhi S.M., Ferguson J.F., Katzmarzyk P.T., Lewis C.E., Owen N., Perry C.K., Siddique J. (2016). Sedentary Behavior and Cardiovascular Morbidity and Mortality: A Science Advisory From the American Heart Association. Circulation.

[9] De Rezende L.F., Rodrigues Lopes M., Rey-López J.P., Matsudo V.K.R., do Carmo Luiz O. (2014). Sedentary behavior and health outcomes: An overview of systematic reviews. PLoS ONE.

[10] De Rezende L.F., Rey-López J.P., Matsudo V.K., do Carmo Luiz O. (2014). Sedentary behavior and health outcomes among older adults: A systematic review. BMC Public Health.

[11] Bullock V.E., Griffiths P., Sherar L.B., Clemes S.A. (2017). Sitting time and obesity in a sample of adults from Europe and the USA. Ann. Hum. Biol..

[12] Salas C., Cristi-Montero C., Fan Y., Durán E., Labraña A.M., Martínez M.A., Leiva A.M., Alvarez C., Aguilar-Farías N., Ramírez-Campillo R. (2016). Ser físicamente activo modifica los efectos nocivos del sedentarismo sobre marcadores de obesidad y cardiometabólicos en adultos [Being physically active modifies the detrimental effect of sedentary behavior on obesity and cardiometabolic markers in adults]. Rev. Med. Chil..

[13] Campbell S.D.I., Brosnan B.J., Chu A.K.Y., Skeaff C.M., Rehrer N.J., Perry T.L., Peddie M.C. (2018). Sedentary Behavior and Body Weight and Composition in Adults: A Systematic Review and Meta-analysis of Prospective Studies. Sports Med..

[14] Hamer M., Stamatakis E., Steptoe A. (2014). Effects of substituting sedentary time with physical activity on metabolic risk. Med. Sci. Sports Exerc..

[15] Mekary R.A., Willett W.C., Hu F.B., Ding E.L. (2009). Isotemporal substitution paradigm for physical activity epidemiology and weight change. Am. J. Epidemiol..

[16] Sardinha L.B., Marques A., Minderico C., Ekelund U. (2016). Cross-sectional and prospective impact of reallocating sedentary time to physical activity on children’s body composition. Pediatric Obes..

[17] Despres J.P. (2015). Obesity and cardiovascular disease: Weight loss is not the only target. Can. J. Cardiol..

[18] Costa R.F.D. (2001). Composição Corporal—Teoria E Prática da Avaliação.

[19] Jefferis B.J., Sartini C., Ash S., Lennon L.T., Wannamethee S.G., Whincup P.H. (2016). Validity of questionnaire-based assessment of sedentary behaviour and physical activity in a population-based cohort of older men; comparisons with objectively measured physical activity data. Int. J. Behav. Nutr. Phys. Act..

[20] Curry W.B., Thompson J.L. (2015). Comparability of accelerometer- and IPAQ-derived physical activity and sedentary time in South Asian women: A cross-sectional study. Eur. J. Sport Sci..

[21] Miller M.R., Hankinson J., Brusasco V., Burgos F., Casaburi R., Coates A., Crapo R., Enright P., Van Der Grinten C.P.M., Gustafsson P. (2005). Standardisation of spirometry. Eur. Respir. J..

[22] Jardim J.R., Oliveira J.A., Nascimento O., Sociedade Brasileira de Pneumologia e Tisiologia (SBPT) (2004). II Consenso brasileiro sobre doença pulmonar obstrutiva crônica (DPOC). J. Bras. Pneumol..

[23] Menezes A.M.B., Perez-Padilla R., Jardim J.B., Muiño A., Lopez M.V., Valdivia G., de Oca M.M., Talamo C., Hallal P.C., Victora C. (2005). Chronic obstructive pulmonary disease in five Latin American cities (the PLATINO study): A prevalence study. Lancet.

[24] Lohman T., Roache A., Martorell R. (1992). Anthropometric Standardization Reference Manual. Med. Sci. Sports Exerc..

[25] WHO (2000). Obesity: Preventing and Managing the Global Epidemic: Report of a World Health Organization Consultation.

[26] Troiano R.P., Berrigan D., Dodd K.W., Masse L.C., Tilert T., McDowell M. (2008). Physical activity in the United States measured by accelerometer. Med. Sci. Sports Exerc..

[27] Brooks A.G., Gunn S.M., Withers R.T., Gore C.J., Plummer J.L. (2005). Predicting walking METs and energy expenditure from speed or accelerometry. Med. Sci. Sports Exerc..

[28] Trost S.G., Way R., Okely A.D. (2006). Predictive validity of three ActiGraph energy expenditure equations for children. Med. Sci. Sports Exerc..

[29] Matthew C.E. (2005). Calibration of accelerometer output for adults. Med. Sci. Sports Exerc..

[30] American College of Sports Medicine (2009). ACSM’s Guidelines of Exercise Testing and Prescription.

[31] American College of Sports Medicine Position Stand (1998). The recommended quantity and quality of exercise for developing and maintaining cardiorespiratory and muscular fitness, and flexibility in healthy adults. Med. Sci. Sports Exerc..

[32] Mekary R.A., Lucas M., Pan A., Okereke O.I., Willett W.C., Hu F.B., Ding E.L. (2013). Isotemporal substitution analysis for physical activity, television watching, and risk of depression. Am. J. Epidemiol..

[33] Galmes-Panades A.M., Varela-Mato V., Konieczna J., Wärnberg J., Martínez-González M.Á., Salas-Salvadó J., Corella D., Schröder H., Vioque J., Alonso-Gómez Á.M. (2019). Isotemporal substitution of inactive time with physical activity and time in bed: Cross-sectional associations with cardiometabolic health in the PREDIMED-Plus study. Int. J. Behav. Nutr. Phys. Act..

[34] Danquah I.H., Pedersen E.S.L., Petersen C.B., Aadahl M., Holtermann A., Tolstrup J.S. (2018). Estimated impact of replacing sitting with standing at work on indicators of body composition: Cross-sectional and longitudinal findings using isotemporal substitution analysis on data from the Take a Stand! study. PLoS ONE.

[35] Grgic J., Dumuid D., Bengoechea E.G., Shrestha N., Bauman A., Olds T., Pedisic Z. (2018). Health outcomes associated with reallocations of time between sleep, sedentary behaviour, and physical activity: A systematic scoping review of isotemporal substitution studies. Int. J. Behav. Nutr. Phys. Act..

[36] Thorp A.A., Owen N., Neuhaus M., Dunstan D.W. (2011). Sedentary behaviors and subsequent health outcomes in adults a systematic review of longitudinal studies, 1996–2011. Am. J. Prev. Med..

[37] Young D.R., Reynolds K., Sidell M., Brar S., Ghai N.R., Sternfeld B., Jacobsen S.J., Slezak J.M., Caan B., Quinn V.P. (2014). Effects of physical activity and sedentary time on the risk of heart failure. Circ. Heart Fail..

[38] Maher C.A., Mire E., Harrington D.M., Staiano A.E., Katzmarzyk P.T. (2013). The independent and combined associations of physical activity and sedentary behavior with obesity in adults: NHANES 2003-06. Obesity.

[39] Murabito J.M., Pedley A., Massaro J.M., Vasan R.S., Esliger D., Blease S.J., Hoffman U., Fox C.S. (2015). Moderate-to-vigorous physical activity with accelerometry is associated with visceral adipose tissue in adults. J. Am. Heart Assoc..

[40] Van Dyck D., Cerin E., De Bourdeaudhuij I., Hinckson E., Reis R.S., Davey R., Sarmiento O.L., Mitáš J., Troelsen J., MacFarlane D. (2005). International study of objectively measured physical activity and sedentary time with body mass index and obesity: IPEN adult study. Int. J. Obes..

[41] García-Hermoso A., Saavedra J.M., Ramírez-Vélez R., Ekelund U., Del Pozo-Cruz B. (2017). Reallocating sedentary time to moderate-to-vigorous physical activity but not to light-intensity physical activity is effective to reduce adiposity among youths: A systematic review and meta-analysis. Obes. Rev..

[42] García-Hermoso A., Martínez-Vizcaíno V., Sánchez-López M., Recio-Rodriguez J.I., Gómez-Marcos M.A., García-Ortiz L., EVIDENT Group (2015). Moderate-to-vigorous physical activity as a mediator between sedentary behavior and cardiometabolic risk in Spanish healthy adults: A mediation analysis. Int. J. Behav. Nutr. Phys. Act..

[43] Coombs N.A., Stamatakis E. (2015). Associations between objectively assessed and questionnaire-based sedentary behaviour with BMI-defined obesity among general population children and adolescents living in England. BMJ Open.

[44] Yoshioka M., Ayabe M., Yahiro T., Higuchi H., Higaki Y., St-Amand J., Miyazaki H., Yoshitake Y., Shindo M., Tanaka H. (2005). Long-period accelerometer monitoring shows the role of physical activity in overweight and obesity. Int. J. Obes..

[45] Ramires V.V., Dumith S.C., Wehrmeister F.C., Hallal P.C., Menezes A.M., Goncalves H. (2016). Physical activity throughout adolescence and body composition at 18 years: 1993 Pelotas (Brazil) birth cohort study. Int. J. Behav. Nutr. Phys. Act..

[46] Lin X., Alvim S.M., Simoes E.J., Bensenor I.M., Barreto S., Schmidt M.I., Ribeiro A.L., Pitanga F., Almeida M.C.C., Liu S. (2016). Leisure Time Physical Activity and Cardio-Metabolic Health: Results from the Brazilian Longitudinal Study of Adult Health (ELSA-Brasil). J. Am. Heart Assoc..

[47] Huang W.Y., Wong S.H., He G., Salmon J.O. (2016). Isotemporal Substitution Analysis for Sedentary Behavior and Body Mass Index. Med. Sci. Sports Exerc..

[48] Aggio D., Smith L., Hamer M. (2015). Effects of reallocating time in different activity intensities on health and fitness: A cross sectional study. Int. J. Behav. Nutr. Phys. Act..

[49] Ekblom-Bak E., Ekblom O., Bergstrom G., Borjesson M. (2016). Isotemporal substitution of sedentary time by physical activity of different intensities and bout lengths, and its associations with metabolic risk. Eur. J. Prev. Cardiol..

[50] Hidalgo K.D., Mielke G.I., Parra D.C., Lobelo F., Simões E.J., Gomes G.O., Florindo A.A., Bracco M., Moura L., Brownson R.C. (2016). Health promoting practices and personal lifestyle behaviors of Brazilian health professionals. BMC Public Health.

[51] Verweij L.M., Coffeng J., van Mechelen W., Proper K.I. (2011). Meta-analyses of workplace physical activity and dietary behaviour interventions on weight outcomes. Obes. Rev. Off. J. Int. Assoc. Study Obes..

[52] Tabak R.G., Hipp J.A., Marx C.M., Brownson R.C. (2015). Workplace social and organizational environments and healthy-weight behaviors. PLoS ONE.

[53] Ryan D.J., Wullems J.A., Stebbings G.K., Morse C.I., Stewart C.E., Onambele-Pearson G.L. (2019). Using isotemporal substitution to predict the effects of changing physical behaviour on older adults’ cardio-metabolic profiles. PLoS ONE.

[54] Turrisi T.B., Bittel K.M., West A.B., Hojjatinia S., Hojjatinia S., Mama S.K., Lagoa C.M., Conroy D.E. (2021). Seasons, weather, and device-measured movement behaviors: A scoping review from 2006 to 2020. Int. J. Behav. Nutr. Phys. Act..

[55] Martins R.C., Reichert F.F., Bielemann R.M., Hallal P.C. (2017). One-year Stability of Objectively Measured Physical Activity in Young Brazilian Adults. J. Phys. Act. Health.

